# Sensory lexicon and aroma volatiles analysis of brewing malt

**DOI:** 10.1038/s41538-022-00135-5

**Published:** 2022-04-11

**Authors:** Xiaoxia Su, Miao Yu, Simin Wu, Mingjuan Ma, Hongxu Su, Fei Guo, Qi Bian, Tianyi Du

**Affiliations:** 1 COFCO Nutrition and Health Research Institute Co., Ltd., Beijing, 102209 China; 2 Beijing Key Laboratory of Nutrition & Health and Food Safety, Beijing, 102209 China; 3 Beijing Engineering Laboratory for Geriatric Nutrition Food Research, Beijing, 102209 China; 4 COFCO Malt (Dalian) Co., Ltd., Dalian, 116113 China; 5 College of Food Science and Nutritional Engineering, China Agricultural University, Beijing, 100083 China

**Keywords:** Nutrition, Quality of life

## Abstract

Malt is an important raw material in brewing beer. With the increasing development of craft beer, brewing malt has contributed diverse colours and abundant flavours to beer. While “malty” and “worty” were commonly used to describe the malt flavour of beer, they are still inadequate. This study focused on developing of a sensory lexicon and a sensory wheel for brewing malt. Here, a total of 22 samples were used for sensory evaluation. The panels identified 53 attributes to form the lexicon of brewing malt, including appearance, flavour, taste, and mouthfeel. After consulting with the experts from the brewing industry, 46 attributes were selected from the lexicon list to construct the sensory wheel. Based on the lexicon, rate-all-that-apply analysis was used to discriminate between six samples of different malt types. The principal component analysis results showed that malt types were significantly correlated with sensory features. To further understand the chemical origin of sensory attributes, partial least squares regression analysis was used to determine the association between the aroma compounds and sensory attributes. According to the colour range and malt types, 18 samples were used for sensory descriptive analysis and volatile compounds identification. Seven main flavours were selected from the brewing malt sensory wheel. 34 aroma compounds were identified by headspace solid phase microextraction gas chromatography-mass spectrometry-olfactometry. According to the partial least squares regression results, the aroma compounds were highly correlated with the sensory attributes of the brewing malt. This approach may have practical applications in the sensory studies of other products.

## Introduction

Beer is a drink that different cultures have appreciated for its variety of ingredients, processing methods, sensory properties, etc^1^. Beer, in particular, has abundant sensory characteristics, including appearance, flavour, taste, and mouthfeel, which can define the overall style of beer and even promote consumption trends^2^. The brewing industry has devoted much effort into the research and development of new technologies and innovations to diversify the flavour of craft beers in response to the increased consumer’s various demands^3–5^. With the increasing production and consumption of craft beer, malt, as an essential raw material in beer brewing, has attracted more attention^6,7^. It has played a key role in endowing beer with diverse colours and abundant flavours^8,9^. Even though “malty” and “worty” were used frequently to describe the malt flavour of beer, they are still inadequate to describe malt’s flavour which contributes a lot to beer^10,11^. Therefore, it is important to study the properties of beer malt alone.

There are two kinds of beer malt; base malts and specialty malts^12^. Base malts are made through a standard process of soaking, germinating, and drying, which provide the basic flavour of beer. Specialty malts are processed on basis of base malts and used for colouring and flavouring. They can be further categorised as High aroma, Biscuit, Caramel, Crystal, Chocolate, Coffee, Black, etc, which contain various flavour characteristics, like roasted, fruity, and caramel^13^. In the past, quality control techniques included visual colour and defects inspection, smelling for off or mould-like aromas, and chewing malt to determine acceptability. However, chewing alone cannot help to accurately describe malt flavour because some flavour active compounds can only be detected after extraction, as would occur in brewing^14^. The Brewing Association surveyed craft brewers and published a white paper “Malting Barley Characteristics for Craft Brewers” that reported malt flavour as a major gap in industry knowledge. Respondents consistently emphasised malt flavour as a priority over other parameters^15^. In response, the industry established several new tools that enable the description of malt flavour. In 2017, the American Society of Brewing Chemists developed and validated the Hot Steep Method, a quick and inexpensive way to extract malt flavour due to its wide accessibility, low material cost, and reproducibility across laboratories. According to Harmonie^16^, the Hot Steep Method has proved to be a highly effective tool, gaining significant popularity in western countries.

Malt flavour is complex because it can be influenced by the chemicals in the malt^17,18^. At the same time, the malt flavour is determined mainly by the malting process, where changes in moisture, temperature, airflow, and pH can affect the final content of non-volatile and volatile compounds^19,20^. The volatile compounds in brewing malts are mainly produced via a series of complex reactions during the germination, drying, and baking stages, such as the Millard reaction, caramelisation, Strecker degradation, and lipid peroxidation and degradation^21,22^. The Maillard Reaction Products (MRPs) are essential contributors to malt flavour and colour^23–25^. Small molecule MRPs, such as pyrazine, furfural and maltol, provide aromas like toasted, nutty, bread, and caramel^25–27^. A work by Sem demonstrated that the concentrations of pyrazine were the highest in roasted malt, such as 2,3-diethyl-5-methylpyrazine and 2-ethyl-3,5-dimethylpyrazine^28^. Furthermore, alcohols, aldehydes, ketones, organic acids, and lipids also contribute to malt flavour^21,27,29^. Dong^30^ indicated that benzaldehyde, benzeneacetaldehyde, hexanol, and ethyl acetate could be the key volatile compounds during the entire malting process.

Sensory evaluation is a crucial method to assess the sensory characteristics of food and beverages^31^. Among sensory evaluation methods, descriptive analysis (DA) has been widely used by researchers to describe the sensory features of food and beverage products^32,33^. In these methods, the sensory properties of the products are described quantitatively by sensory panel^34,35^. There has been a growing application of a series of novel sensory profiling methods, such as check all-that-apply (CATA), and rate-all-that-apply (RATA), which enables the use of untrained panellists or even consumers to obtain sensory profiles of food products^36,37^. However, the key to using those novel approaches is establishing an appropriate sensory lexicon based on panel input. Lawless and Civille^38^ provided an overview of the essential elements of a sensory lexicon, which should include a list of relevant products, along with all the attributes and their definitions and references. Recently, sensory wheels have been developed for a variety of beverages and foods, including chocolate^39^, Rakı^40^, potato cultivar^41^, soy sauce^42^, and coated tablets^43^. The attributes in a sensory wheel can be used to standardise training and aid education and discussion. Sensory wheels can also serve as a communication tool among industry members, including producers, retailers, exporters, importers, industry professionals, and consumers^40^.

Until now, there have been no detailed studies on the sensory properties of brewing malt. This study aimed to develop a sensory lexicon for brewing malt by sensory panel. Based on the sensory lexicon, the primary descriptors can be selected and visualised in the form of a sensory wheel. With the identification of the aroma-active compounds by gas chromatography-olfactometry (GC-O), the relationships between the aroma compounds and flavour attributes can be further explored.

## Results and discussion

### Lexicon and sensory wheel

Lexicon development included the following steps: identifying the broad product scope, generating the terms to characterise the products, and developing a final list of descriptors with references^38^. The 22 brewing malt samples represented the most likely sensory attributes to develop a brewing malt lexicon. In the beginning, the assessors generated a total of 214 sensory terms. Then, with further discussion, similar terms were combined by the consensus, and 92 preliminary descriptive terms were identified (Table 1). Some of the preliminary terms were then eliminated if their mention frequency was less than 5% before the final brewing malt lexicon was determined^39,42^. Finally, 53 attributes and their definitions and references were developed for the brewing malt lexicon. The final attributes list is shown in Table 2 and their definitions and references are shown in Supplementary Table 1. 53 attributes were then grouped into different categories, which was agreed upon by the panel, sensory research team, and the experts from the brewing industry. Overall, there were 9 categories in the lexicon, including 17 appearance attributes, 27 flavour attributes, and 9 taste and mouthfeel attributes.

**Table 1:** Preliminary list of the descriptive terms generated by the sensory panel for the brewing malts and their usage frequency.

Appearance	F%	Flavour	F%	Flavour	F%	Taste/mouthfeel	F%
Black	20%	Over burnt	4%	Sugar cane	14%	Sweet	66%
Brown black	13%	Cinnamon	1%	Fruity	35%	Sweet aftertaste	5%
Dark brown	8%	Smokey	4%	Red dates	23%	Yeasty sour	4%
Brown	6%	Clove	5%	Vanilla	5%	Wine sour	8%
Light brown	5%	Roasted	25%	Corn	15%	Espresso sour	8%
Coppery	10%	Smoked	35%	Wheatmeal	1%	Sour	55%
Dark amber	8%	Ovaltine	2%	Corn flakes	1%	Radix isatidis	4%
Amber	9%	Rice crust	4%	Honey	27%	Black coffee bitter	6%
Light straw	15%	Maillard	4%	Malty	38%	Bitter	45%
Lemon yellow	18%	Baking	56%	Biscuit	21%	Balancing	4%
Transparent	15%	Black chocolate	18%	Cereal	10%	Smooth	4%
Semitransparent	5%	Burnt	37%	Barley tea	9%	Mouthwatering	2%
Translucent	15%	Coffee	15%	Sprouts	1%	Lightness	31%
Semitranslucent	6%	Roasted nut	25%	Green bean	3%	Astringency	25%
Opaque	25%	Toast	24%	Dry grass	6%		
Consistency	14%	Cake	1%	Cucumber	6%		
Cup-hanging	8%	Brown sugar	2%	Plantule	10%		
		Caramel	46%	Grass	15%		
		Roasted sweet potato	30%	Green	33%		
		Toffee	10%	Stale	2%		
		Molassess	17%	Woody	1%		
		Sunflower seed	2%	Solvent	1%		
		Walnut	1%	Soil	1%		
		Peanut	3%	Black tea	1%		
		Nutty	30%	Metallic	1%		
		Raw nutty	12%	Green tea	2%		
		Almond	9%	Dirt	2%		
		Hazel	15%	Dairy	1%		
		Floral	2%	Papery	2%		
		Dimethylsulfide	4%	Cork	4%		
		Apple	3%

**Table 2:** Attributes for the brewing malts.

Categories of Attributes	Attributes	Categories of Attributes	Attributes
Colour	Lemon yellow	Smoky	Clove
	Light straw		Smoked
	Amber		Roasted
	Dark amber	Baking	Coffee
	Coppery		Black chocolate
	Light brown		Toast
	Brown		Roasted nut
	Dark brown		Burnt
	Brown black	Caramel	Caramel
	Black		Toffee
Transparency	Transparent		Caramelised sweet potato
	Semitransparent		Molasses
	Translucent		Honey
	Semitranslucent	Nutty	Almond
	Opaque		Hazel
Others	Sweet		Raw nuts
	Sweet aftertaste	Fruity	Sugar cane
	Wine sour		Vanilla
	Espresso sour		Red dates
	Sour		Corn
	Black coffee bitter	Grain	Biscuit
	Bitter		Cereal
	Lightness		Barley tea
	Astringency	Green	Grass
	Consistency		Cucumber
	Cup-hanging		Plantule
			Dry grass

**Fig. 1:**
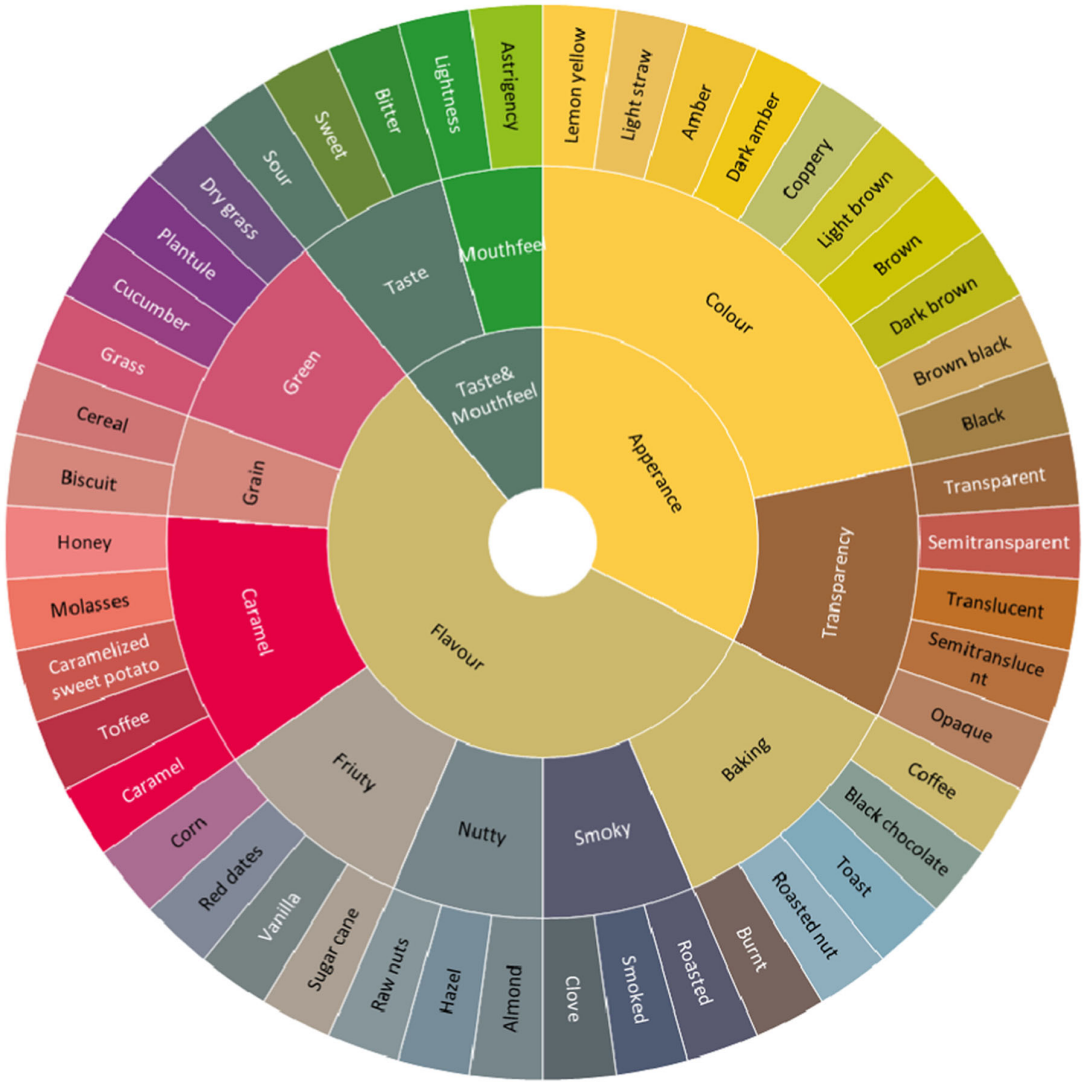
Sensory wheel of brewing malts. 46 specific attributes of brewing malt were illustrated in a three-circle sensory wheel, including 15 appearance attributes, 26 flavour attributes and 5 taste and mouthfeel attributes.

A sensory wheel was generated according to the final attribute list from the brewing malt lexicon. These attributes were grouped to constitute different categories according to the inputs of the sensory research team. After consulting with the experts from the brewing industry and discussing among the sensory research team, 7 terms were dropped from the lexicon list (Table 2). A total of 46 specific attributes of brewing malt were illustrated in a three-circle sensory wheel, including 15 appearance attributes, 26 flavour attributes, and 5 taste and mouthfeel attributes (Fig. 1). The descriptors that made up the outer circle were the specific attributes, while the secondary descriptors that grouped certain attributes made up the second tier. The inner circle contained three primary sensory modalities: appearance, flavour, and taste and mouthfeel. In this study, a lexicon and sensory wheel for brewing malt were developed for the first time. The sensory lexicon and the sensory wheel specified a set of terms that defined the general properties of brewing malt for industrial and academic needs.

### RATA analysis based on the flavour and taste attributes of brewing malt Lexicon

**Fig. 2:**
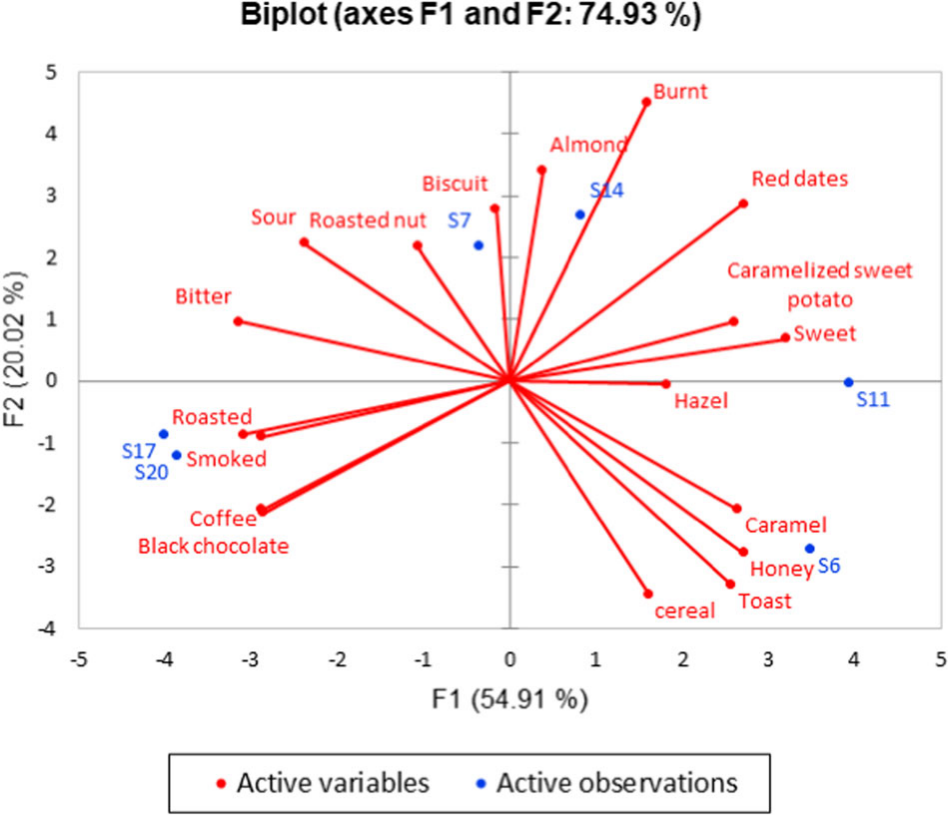
Principal component analysis for six brewing malt samples. Correlations between 18 sensory attributes and specialty malts (*p* < 0.05).

In order to validate whether the descriptors in the sensory wheel could help to distinguish between different malt samples, RATA analysis was conducted. Six brewing malt samples were selected from six different categories (High aroma, Biscuit, Caramel, Crystal, Chocolate, and Black) from Table 3. Fifteen attributes of flavour and 3 attributes of taste were chosen based on the common agreement of the laboratory panel. The attributes were mainly derived from flavour and taste, including toast, caramelised sweet potato, honey, red dates, cereal, caramel, biscuit, roasted, smoked, burnt, roasted nut, hazel, almond, black chocolate, coffee, sour, sweet, and bitter. The details are given in Supplementary Table 2. Principal component analysis (PCA) was used to evaluate the sensory data gathered from the six brewing malt samples by the Laboratory Panel. The results are shown in Fig. 2, and the variance is explained by the two principal components. The first principal component explained 54.91% of the variance, while the second principal explained 20.02%. It was shown that there were significant differences in the flavour attributes of the six brewing malt samples, which could be divided into three classes by PCA. Two of the samples, S6 and S11, were similar, displaying lower intensities of roasted, smoked, coffee, and black chocolate flavours and higher intensity levels of caramel, honey, and toasted flavours. The S7 and S14 samples were also similar, with prominent burnt, almond, biscuit, and roasted nut flavours. Furthermore, similarities were evident between the two remaining samples, S17 and S20, with roasted, smoked, coffee, and black chocolate flavours. Coghe^21^ indicated that a trained tasting panel detected a higher intensity of bitter and burnt flavours as the colour of the malt increased while the sweet flavour notes decreased. Chocolate and Black malt were processed with higher baking temperature and baking time than the other four malts, maximising the bitter, roasted, and smoky flavours. Based on the lexicon, RATA was used to discriminate between samples of different malt types (High aroma, Biscuit, Caramel, Crystal, Chocolate, and Black). The results of PCA show that malt type was significantly correlated with the sensory features.

**Table 3:** Overview of the brewing malt samples.

Sample code	Sample name	Malt type	Brand	Colour (EBC)
S1^☆○^	BaseASC	Base	Puremalt	3–6
S2^☆^	BaseABA	Base	Puremalt	3–6
S3^☆^	BaseCMET	Base	Puremalt	3–6
S4	Munich	Munich	Puremalt	4.3–8.1
S5^☆^	High aroma I	High aroma	Puremalt	27
S6^★○^	High aroma II	High aroma	Puremalt	42
S7^★☆^	Biscuit І	Biscuit	Puremalt	80
S8^☆^	Biscuit II	Biscuit	Puremalt	89
S9^☆^	Honey Biscuit	Biscuit	Swan	80–90
S10^☆^	CaraRed	Caramel	Weyermann	40–50
S11^★☆○^	Caramel	Caramel	Puremalt	100–120
S12^☆^	CaraMunich Type 2	Caramel	Weyermann	110–130
S13^☆^	Crystal I	Crystal	Puremalt	140
S14^★☆○^	Crystal II	Crystal	Puremalt	323
S15^☆^	Crystal III	Crystal	Malteurop	150
S16^☆^	Chocolate I	Chocolate	Puremalt	435
S17^★○^	Chocolate II	Chocolate	Puremalt	643
S18^☆^	Chocolate III	Chocolate	Briess	950
S19^☆^	Coffee	Coffee	Puremalt	400
S20^★☆○^	Black І	Black	Puremalt	1404
S21^☆^	Black II	Black	Puremalt	865
S22	Carafa Type 3	Black	Weyermann	1300–1500

★Sample Code with^★^ were researched by laboratory panel in the rate-all-that-apply analysis

○Sample Code with^○^ were researched by gas chromatography -olfactometry analysis

☆Sample Code with^☆^ were researched by laboratory panel in the descriptive analysis and headspace solid phase microextraction gas chromatography-mass spectrometry analysis

### Analysis of the aroma-active compounds

18 selected brewing malt samples, representing different colours and types (Base 3, High aroma 2, Biscuit 3, Caramel 2, Crystal 3, Chocolate 2, Coffee 1, and Black 2), were selected from Table 3 and determined by headspace solid phase microextraction gas chromatography-mass spectrometry (HS-SPME-GC-MS). A total of 76 volatile compounds were identified by HP-5MS columns, including 23 aldehydes, 15 heterocycles, 15 esters, 7 alcohols, 7 ketones, 2 phenols, 3 olefins, and 4 others (Supplementary Table 3). 7 brewing malt samples were selected from Table 3, representing 7 different categories (Basic, High aroma, Biscuit, Caramel, Crystal, Chocolate, and Black). GC-O analysis was used to determine the overall odour of aroma-active compounds. As shown in Table 4, 34 aroma compounds were detected in the samples, including 16 aldehydes, 11 heterocycles, 5 alcohols, 1 ketone, and 1 phenol. The results show that base malts and specialty malts had a diverse profile of aroma compounds. The content and quantity of aroma compounds in base malts were the lowest, and aldehydes were the most important. In contrast, the aroma compounds of specialty malts were more abundant and were affected by the malting process.

**Table 4:** The aroma compounds in brewing malts.

No.	Compounds	Aroma descriptors	Significance^a^	No.	Compounds	Aroma descriptors	Significance^a^
1	Isobutyraldehyde	Baked	Yes	34	2-Isopropyl-5-methyl-2-hexenal	Milk chocolate	Yes
2	Isovaleraldehyde	Nutty	Yes	35	Trans-2-nonenal	Green	Yes
3	2-Methylbutyraldehyde	Baked	Yes	36	Maltols	Caramellic	Yes
4	Valeraldehyde	Nutty	Yes	38	2-Methyl-3-isobutylpyrazine	Sugar syrup	Yes
6	Hexanal	Green	Yes	42	1-Furfurylpyrrole	Nutty, cream	Yes
8	Furfural	Red dates	Yes	43	Decyl aldehyde	Crisps	Yes
9	Furfuryl alcohol	Coffee	Yes	44	2-Propylheptan-1-ol	Rice bran	Yes
15	2-Acetylfuran	Earthy	Yes	49	2-Phenyl-2-butenal	Digestive biscuit, burnt	Yes
18	Benzaldehyde	Plantule	Yes	50	4-Ethyl-2-methoxyphenol	Smokey, spicy	Yes
19	5-Methyl furfural	Coffee, chocolate	Yes	52	Trans,Trans-2,4-Decadien-1-al	Earthy, green	Yes
20	1-Octen-3-ol	Green	Yes	56	4-Methyl-2-phenyl-2-pentenal	Cerieal	Yes
21	2-Pentylfuran	Earthy	Yes	59	Geranylacetone	Green	Yes
22	2-Ethyl-6-methylpyrazine	Baked	Yes	61	1-Dodecanol	Earthy	Yes
24	2-Pyrrolylcarboxaldehyde	Coffee	Yes	62	Cocal	Fruity	Yes
26	2-Ethylhexanol	Fruity	Yes				
28	Phenylacetaldehyde	Honey, sweet potato	Yes				
29	Trans-2-octenal	Flowery, green	Yes				
30	2-Acetyl pyrrole	Burnt	Yes				
31	3,5-Dimethyl-2-ethylpyrazine	Nutty, baked	Yes				
33	Nonanal	Honey	Yes				

^a^The contents of aroma compounds in malt samples are significantly different (*p* < 0.05) using Fisher’s least significant difference (LSD) tests

The volatile compounds in brewing malt are mainly produced by the Maillard reaction, caramelisation, Strecker degradation, and lipid peroxidation degradation^21,22^. Both caramelisation and the Maillard reaction are non-enzymatic Browning reactions, giving appealing flavours to malt under high temperature. Caramelisation is a process during which carbohydrate compounds dehydrate and degrade, producing volatile aldehydes and ketones. Overall, seven ketones were identified in the 18 brewing malts. It should be noted that geranylacetone (green) was a featured flavour compound found in the specialty malts but not in base malts. On the other hand, the Maillard reaction involves the violent collision and recombination of amino acids and reducing sugars in high-temperature conditions, yielding hundreds of compounds^24,44^. Base malts had the lowest degree of the Maillard reaction and thus produced only a small number of heterocyclic compounds. In contrast, the Maillard reaction of specialty malts produced more heterocyclic compounds. During the heating process, many N-heterocycles, O-heterocycles were produced, such as pyrazine, pyrrole, and furan. Studies have shown that MRPs are important compounds in malts, giving off roasted, coffee, nutty, caramel flavours^21,25^. Caramel malt and Crystal malt are prepared by multi-step roasting of green malt. Caramel malt and Crystal malt presented burnt flavour^45^, mainly from oxygen-containing heterocyclic compounds produced during baking^46^. Six O-heterocycles were identified in caramel and crystal malt samples, and five of them were aroma compounds, including furfural (red dates), furfuryl alcohol (coffee), 2-acetylfuran (earthy), 2-pentylfuran (earthy), and 5-methyl furfural (coffee and chocolate). In addition, furfural was found in all specialty malts samples and its content increased exponentially with the deepening of the malt colour. These results suggest that the furfural formation might require higher thermal energy than the other compounds. The High Aroma, Biscuit, chocolate, and black malt are all processed from base malts. As the baking process progressed, the EBC value gradually increased, while the quantity and content of MRPs also increased. Nitrogen-containing heterocyclic compounds such as pyrazine and pyrrole contributed significantly to the aroma of the dark malt. Nine N-heterocycles were identified in chocolate, coffee, and black malt samples, and six of them were aroma compounds, which were 2-ethyl-6-methylpyrazine (baked), 3,5-dimethyl-2-ethylpyrazine (nutty and baked), 2-methyl-3-isobutylpyrazine (sugar syrup), 2-pyrrolylcarboxaldehyde (burnt), and 1-furfurylpyrrole (nutty and cream). Aldehydes, alcohols, ketones, and esters are mainly related to lipid oxidation. Isovaleraldehyde (nutty), hexanal (green),benzaldehyde (plantule), trans-2-octenal(green), nonanal (honey), and decyl aldehyde (crisps) were identified in the 18 brewing malts. Basic malt contained the most esters, which could be gradually broken down during baking, so very few lipids were left in dark malt. In the previous research, trans-2-nonenal was found to be a vital aroma component in barely, malt, and beer^47^, which was mainly found in base malts. 2-methylbutyraldehyde (baked), and Cocal (fruity) were only identified in the specialty brewing malts. Maltols (caramellic) are crucial alcohol components. While this substance is a prominent aroma compound in the Caramel and Crystal Malt samples, they are also present in the Coffee, Chocolate, and Black malts.

### The relationships between the main flavour attributes and aroma compounds

In this study, the Laboratory Panel using DA evaluated the selected 18 samples (Supplementary Table 4). Seven categories of flavour attributes in the sensory wheel were selected for evaluation: baking, smoky, nutty, fruity, caramel, grain, and green.

**Fig. 3:**
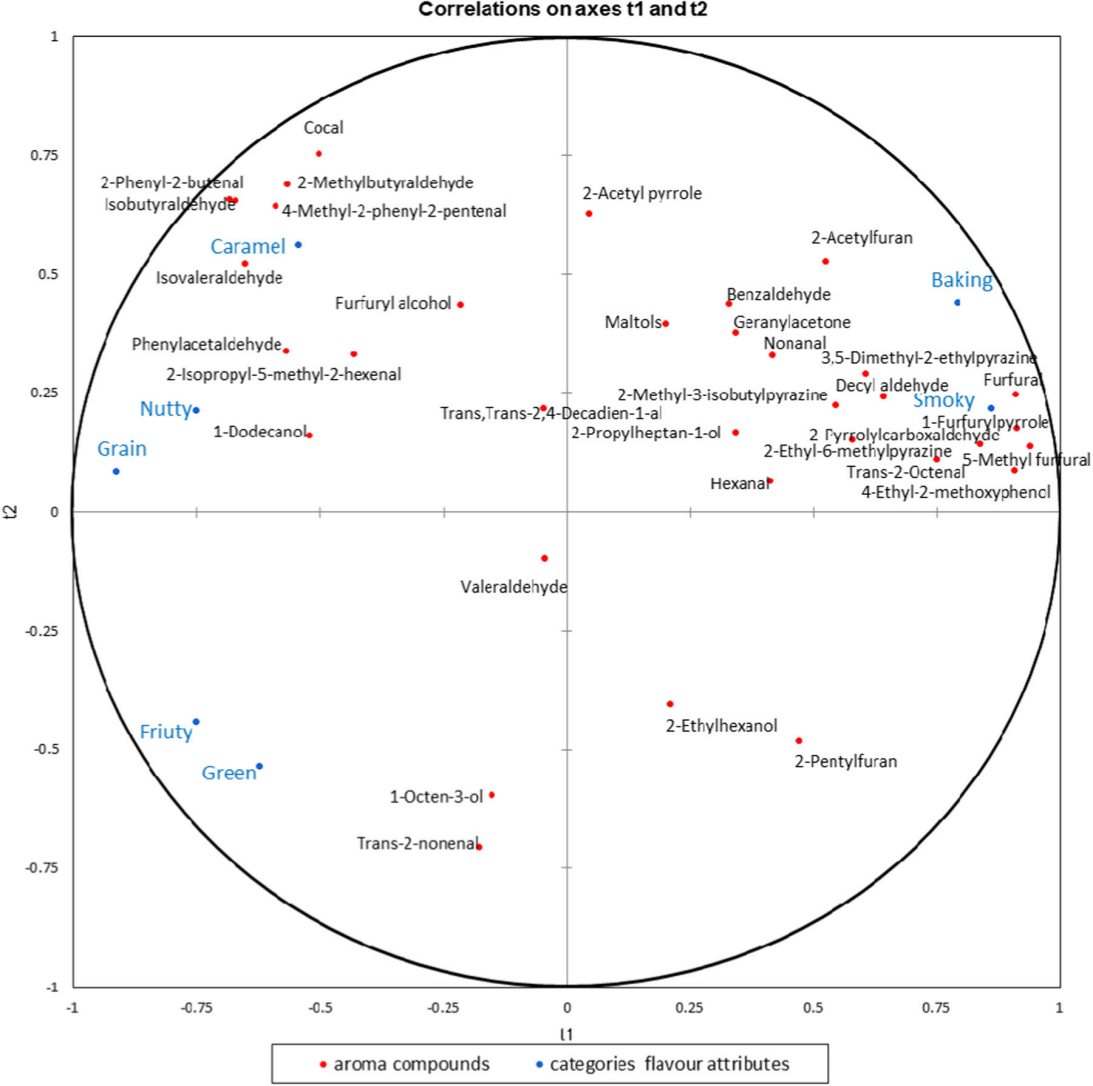
Partial least squares regression model analysis for 18 brewing malt samples. Correlations between seven categories of flavour attributes and the aroma compounds (*p* < 0.05).

The brewing malt characteristics (both sensory and chemical) were expected to be interdependent and therefore it was difficult to analyse them independently. Therefore, multivariate statistical analysis was employed to establish mathematical models to clarify the relationship between the aroma compounds and the sensory properties^48^. To further understand the chemical origin of the sensory attributes, partial least squares (PLS) regression analysis was used to determine the association between the categories of flavour attributes (*y* variables, *n* = 7) and the significantly different aroma compounds (*x* variables, *n* = 34) in the 18 brewing malt samples. The performance of the PLS model was determined using cross-validation parameters. *R*^*2*^*Y* and *Q*^*2*^ represented the discussed variance and the predictive capability of the model, respectively. As a function for the goodness of fit, greater *R*^*2*^*Y* values indicated better modelling^49^. As an indicator of the prediction ability of the model, *Q*^*2*^ value >0.5 was admitted for good predictability^50^. Taking into account that a large discrepancy between *R*^*2*^*Y* and *Q*^*2*^ indicated an overfitting of the model through the use of too many components, the difference between *R*^*2*^*Y* and *Q*^*2*^ should be lower than 0.3^50^. The concentration of the aroma compounds and the rating of categories of flavour attributes are shown in the correlation loading plot (Fig. 3). The value of *R*^*2*^*Y* was 0.728, and *Q*^*2*^ was 0.620, showing good fitness and predictability of the model. Therefore, the aroma compounds plotted in the vicinity of the sensory attributes were positively associated with those attributes.

The variable importance for projection (VIP) values were calculated from the PLS regression model of the sensory attributes and the aroma compounds (*p* < 0.05) of the 18 brewing malt samples. The VIP scores were above 1 for the 13 aroma compounds, which were furfural, 5-methyl furfural, 4-ethyl-2-methoxyphenol, 1-furfurylpyrrole, 2-pyrrolylcarboxaldehyde, trans-2-octenal, 3,5-dimethyl-2-ethylpyrazine, isovaleraldehyde, 2-ethyl-6-methylpyrazine, 2-phenyl-2-butenal, 4-methyl-2-phenyl-2-pentenal and 1-dodecanol. The presence of these aroma compounds was considered the primary reason for the flavour differences in brewing malt. Moreover, the “baked” and “smoky” flavours were associated with furfural, 1-furfurylpyrrole, 5-methyl furfural, 4-ethyl-2-methoxyphenol, 2-pyrrolylcarboxaldehyde, trans-2-octenal, 3,5-dimethyl-2-ethylpyrazine, decyl aldehyde, 2-methyl-3-isobutylpyrazine, and 2-ethyl-6-methylpyrazine. The heterocycles were the most important volatile constituents in the brewing malts. Their presence generally contributed to the roasted flavour notes of the baking products, which was consistent with the results of the PLS regression. The “caramel” flavour was related to cocal, 2-methylbutyraldehyde, isobutyraldehyde, 4-methyl-2-phenyl-2-pentenal, and furfuryl alcohol. The “nutty” and “grain” flavours were connected with 2-isopropyl-5-methyl-2-hexenal, phenylacetaldehyde, and 1-dodecanol. Previously, phenylacetaldehyde was considered as one of the key odorants during the whole malting process^22^. The “green” and “fruity” flavours displayed a close correlation with 1-octen-3-ol and trans-2-nonenal. In previous work by Akira^51^, it was demonstrated that 1-octen-3-ol and trans-2-nonenal contributed to the green note in malt whisky.

PLS regression analysis identified that the aroma compounds were highly correlated with the sensory attributes of the brewing malt. This was an essential strategy for identifying the critical aroma compounds that accounted for sensory differences and can be applied to optimise the parameters of malt brewing and beer production processes to improve product quality and flavour characteristics.

## Methods

### Experimental design

First, this study organised two sensory panels (Industry Panel and Laboratory Panel) in order to obtain a sensory lexicon for brewing malt. Next, a sensory wheel was constructed by selecting the most relevant terms according to their mention frequency. Using the reduced list of the sensory lexicon, the laboratory panel evaluated a total of six brewing malt samples with the rate-all-that-apply (RATA) method. Ultimately, sensory evaluation and headspace solid phase microextraction gas chromatography-mass spectrometry-olfactometry (HS-SPME-GC-MS-O) were used to analyse the relationship of flavour attributes and volatile compounds in 18 brewing malts.

### Malt samples

When developing the sensory descriptors, it was important to cover the full range of the sensory profile in order to capture as many sensory descriptors as possible^40^. A total of 22 commercial brewing malt samples (denoted S1–S22) were selected as representative samples, which were gathered from 9 malt types and covered a wide colour scale (3 EBC to 1500 EBC) in order to explain all potential sensory attributes (Table 3). The malt samples were obtained from malt manufacturers or retail outlets, while their product classification information was taken from each one of the producers. Samples were stored at a constant temperature of 25 °C. For volatile compounds analysis and sensory analysis, wort samples were prepared according to the Hot Steep Method of the American Society of Brewing Chemists^16^. During a sensory evaluation, the wort samples were presented in a randomised order and served at 25 °C in odourless transparent cups with an adequate volume of 30 mL. There was a one-minute break between each sample to prevent fatigue. Water and plain crackers were available for palate cleansing.

### HS-SPME-GC-MS-O analysis of volatile compounds

Volatile compounds in the wort samples were detected using HS-SPME-GC-MS. 5 mL of the wort and 150 μL of 1,2,3-trichloropropane (10 mg/L in methanol, internal standard) were placed in a 20 mL glass vial sealed with an aluminium cover and Teflon septum. The samples were equilibrated for 20 min at 60 °C in the vials, and a 50/30 μm DVB/CAR/PDMS fibre (Supelco Ltd., Bellefonte, PA) was exposed to the sample headspace at 60 °C for 40 min. After extraction, the fibre was desorbed in a splitless inlet at 250 °C for 5 min. An Agilent 7890B/5977 A GC-MS (Agilent Technologies Inc., Santa Clara, CA) was used for GC-MS analysis, equipped with HP-5MS chromatographic columns (30 m × 0.25 mm i.d., 0.25 μm film thickness, J&W Scientific, Folsom, CA). Helium was used as the carrier gas, at a constant current of 1.20 mL/min. The GC programme was as follows: The original oven temperature was set at 35 °C for 5 min, gradually increased to 85 °C at a rate of 10 °C/min and held for 1 min, then raised to 200 °C at 4 °C/min and held for 3 min and finally raised to 200 °C at 10 °C/min and held for 3 min. The electron energy of MSD was 70 eV, the temperature of the ion source was 250 °C, and the scanning range was m/z 40–500. The volatile compounds were identified based on the NIST 2014 (National Institute of Standards and Technology, Gaithersburg, MD) and the retention index. Each examination was repeated three times. The relative content of each volatile compound was calculated according to the normalised scanning total ion current peak area using the internal standard, and the final result was the average value of the three replicate calculations.

Aroma-active compounds in the wort samples were directly detected by the sniffing port of the GC-MS-O (Sniffer 9000, Brechbuhler AG, Switzerland). The analysis conditions were the same as those for the GC-MS using the HP-5MS column, while the temperature of the sniffing port was set at 200 °C. During the GC operation, the panellists placed their noses close to the sniffer port and then recorded the smells. The compounds identified by more than three panellists were selected as flavour compounds for further analysis.

### Panel

Sensory studies were performed by two independent groups, namely, the Industry Panel and the Laboratory Panel. The Industry Panel comprised 8 assessors (6 females and 2 males; aged range 26–40 years for both genders), with more than five years of both industrial and sensory analysis experience. The group consisted of R&D specialists from the Research and Development Centre of the COFCO Malt (Dalian) Co. Ltd and the sensory research team of COFCO NHRI Co. Ltd. Members of the R&D department were selected according to their regular participation in sensory evaluations for product development and quality control in industrial production. The sensory research team also regularly participated in sensory analysis research at the COFCO Sensory and Flavour Lab (CSFL). The Laboratory Panel comprised 15 assessors (12 females and 3 males; aged range 26–50 years for both genders), with more than two years of sensory analysis experience. They were recruited in compliance with ISO standards and were selected based on their abilities to identify and describe differences in malt samples. The study was reviewed and approved by the COFCO NHRI Co. Ltd. All assessors had provided informed consent before gathering in the CSFL testing room. For each sensory evaluation, the experiment was finished in separate compartments, under about 25 °C temperature and around 50% relative humidity. The panellists were instructed to observe, sniff, and taste the sample as many times as needed. The definitions of appearance (all the visible attributes of a substance or object), flavour (a complex combination of the olfactory, gustatory and trigeminal sensations perceived during tasting), taste, and mouthfeel (mixed experience derived from the taste organ or sensations in the mouth that relate to physical or chemical properties of a stimulus) were explained thoroughly to the panel^52^.

### Development of the sensory lexicon and formation of the sensory wheel

The panel leader welcomed the assessors and explained that the aim of the experiments was to determine the sensory descriptors for brewing malts. The panel leader made sure to keep everyone engaged and focused on the task. Both of the sensory groups completed seven sessions (2 h each) in the sensory room. The first three sessions involved term generation based on all wort samples. In each session, seven or eight samples were served to the assessors. Panellists were individually presented with the samples and asked to write down the sensory attributes they found. Appearance, flavour, taste, and mouthfeel were evaluated based on their sensory perception. To describe the samples, they were instructed not to use hedonistic or quantitative terms, such as good, well, just right, and so forth. Assessors specified the attributes, definitions, and references through a consensual process^38,53^. In order to finalise the list of descriptive terms, the following sessions focused on attribute alignment with the use of references. The panel leader led several group discussions to reach a consensus on terminology. During the discussion, the panellists explained to each other the terms they would use to describe the samples, shared their understandings, and produced unified expressions. Finally, combining terms from the two different sensory groups, a total of 214 attributes were defined.

After that, the terms were reviewed and rationalised by assessors. The terms and references understood by more than two-thirds of the panellists were adopted as brewing malt descriptors^42^. Then, the descriptors with the same or similar meaning and redundant terms were eliminated. Only consensus attributes directly related to the samples were included in the preliminary descriptor list (*n* = 92). For practical purposes, the list was further consolidated based on how frequent the panellists used the terms. Attributes with low mention frequency (<5%) were removed^38,39^. The final list of the descriptors for brewing malt was then used to build the sensory wheel. They were divided into different categories based upon the inputs of the sensory research team. The sensory wheel was constructed using XLSTAT software.

### Descriptive analysis of brewing malt samples

In the last decades, researchers have developed a number of rapid profiling methods, among which RATA was reported to be an effective method for providing the sensory characterisation of food products^54,55^. Recently, RATA has been successfully applied to differentiate the visual appearance of milk powders using trained sensory panels^37^. Vannier^56^ indicated that an efficient sensory profiling could be achieved with about 20 attributes. In this study, the Laboratory Panel applied RATA to evaluate six malt samples selected from six different categories (High aroma, Biscuit, Caramel, Crystal, Chocolate, and Black) after training. The panellists completed three sessions (2 h each). During the first session, a total of 18 attributes from the brewing malt sensory wheel were selected and agreed upon by the panellists. The second session was to evaluate six malt samples using RATA, and in the final session, the procedure above was repeated. Reference standards were prepared to aid the assessors. For each sample, the panellists were asked to respond “yes” to the attribute(s) they considered appropriate to describe the sample, and then rate the intensity of the attribute(s), using a 5-point scale (“very weak”, “weak”, “medium”, “strong”, and “very strong”). When testing, the absence of the attribute was scored as zero (0) and the intensity levels of the presence attributes were scored as 1, 2, 3, 4, or 5. The order in which the terms were presented was random for each product and participant. The samples were evaluated in duplicate to obtain reliable, consistent results.

### Correlation analysis of the flavour attributes and the aroma compounds

Meanwhile, according to different categories (Base 3, High aroma 2, Biscuit 3, Caramel 2, Crystal 3, Chocolate 2, Coffee 1, and Black 2), 18 brewing malt samples were selected from Table 3. The Laboratory Panel used descriptive analysis (DA) to evaluate the samples.

Panellists completed a total of six sessions and assessed six samples per session, with two replicates for each malt sample. The seven categories of flavour attributes of the sensory wheel were selected as the evaluation attributes. The attribute intensity was scored using the continuous 5-point scale, ranging from 0 (lowest intensity) to 5 (highest intensity). To understand the chemical origin of the flavour attributes, PLS regression was employed to analyse the correlation between the categories of flavour attributes and the aroma compounds of 18 brewing malt samples.

### Statistical analysis

Analysis of variance (ANOVA) was used for RATA and DA sensory data to determine the differences between the samples. The PCA was conducted to analyse the relationship between the malt samples and the sensory attributes. PLS regression was conducted to find out any correlation between the categories of flavour attributes and the aroma compounds. ANOVA, PCA, and PLS regression were run by XLSTAT software (Version 2019). Panel performance was monitored using PanelCheck Software (Version 1.3.2).

### Reporting summary

Further information on research design is available in the Nature Research Reporting Summary linked to this article.

## Supplementary information

Supplementary data

Reporting Summary

## Data Availability

The authors declare that all data supporting the findings of this study are available within the paper and supplementary information.
