# Supplementary material for: Sensory lexicon and aroma volatiles analysis of brewing malt
Source: NPJ Sci Food. 2022 Apr 11;6:20. doi: 10.1038/s41538-022-00135-5 (PMC9001694; doi:10.1038/s41538-022-00135-5)
Supplement: Supplementary file 1 — Supplementary data [file 41538_2022_135_MOESM1_ESM.pdf]

Supplementary Table 1. Attributes and references for the brewing malts.

Categories of Attributes	Attributes	Definitions	References
Colour	Lemon yellow	A light yellow colour	AIH SRM Number Scale 1-2
	Light straw	The colour of dried, yellow stems of crops darker than lemon yellow	AIH SRM Number Scale 3-6
	Amber	A transparent, yellowish-brown	AIH SRM Number Scale 7-9
	Dark amber	A transparent, light-brown with little yellowish	AIH SRM Number Scale 10-12
	Coppery	A rich coppery colour	AIH SRM Number Scale 13-18
	Light brown	A light brown colour	AIH SRM Number Scale 19-28
	Brown	The colour of chocolate or soil	AIH SRM Number Scale 29-34
	Dark brown	Darker brown colour	AIH SRM Number Scale 35-37
	Brown black	The black colour with a little brown	AIH SRM Number Scale 38-39
	Black	The colour of coal	AIH SRM Number Scale 40+
Transparency	Transparent	Allowing the sight to see through it	Water
	Semitransparent	Imperfectly transparent	Green tea
	Translucent	Allowing light, but not detailed shapes, to pass-through	Black tea
	Semitranslucent	Imperfectly translucent	Americano
	Cup-hanging	The fall rate of the liquid trace after shaking	Wine
Smoky	Clove	A flavour reminiscent of spicy, slightly minty, floral notes, associated with clove	Dry cloves
	Smoked	A flavour reminiscent of smoky, slightly spicy, salty notes	Smoked wood
	Roasted	A flavour reminiscent of roasted food flavour	Burnt charcoal
Baking	Coffee	A flavour reminiscent of roasted, bitter with coffee	Coffee bean
	Black chocolate	A flavour reminiscent of bitter, sweet with black chocolate	70% black chocolate
	Toast	A flavour reminiscent of roasted bread, associated with bakery	Croissant
	Roasted nut	A flavour reminiscent of roasted peanut/sunflower seeds	Roasted nut essence
	Burnt	A flavour reminiscent of burnt nuts, such as peanut/ walnuts	burnt walnuts
Caramel	Caramel	A flavour reminiscent of rich, sweet, buttery notes associated with caramel	Caramel

		flavours	
	Toffee	A flavour reminiscent of sweet, caramelized, milky, associated with toffee flavours	Toffee candy
	Caramelized sweet potato	A flavour reminiscent of roasted sweet potato	Roasted sweet potatoes
	Molasses	A flavour reminiscent of caramelized sugar, acrid and molasses-like notes, associated with syrup flavours	Molasses
	Honey	A flavour reminiscent of honey	Honey solution
Nutty	Almond	A flavour reminiscent of woody and cherry-like notes, associated with almond	Almond
	Hazel	A flavour reminiscent of nutty, earthy, oily notes, associated with hazelnut flavours	Hazelnut
	Raw nuts	A flavour reminiscent of fresh nutty notes, associated with raw peanut/sesame/sunflower seeds flavours	Raw peanut
Fruity	Sugar cane	A flavour reminiscent of fresh fruity sweet notes, associated with sugar cane	Sugar cane
	Vanilla	A flavour reminiscent of sweet, spicy, musk ,and vanilla notes associated with vanilla flavours	Vanilla essence
	Red dates	A flavour reminiscent of red dates	Red dates
	Corn	A flavour reminiscent of fresh, sweet, green, associates with corn	Cooked fresh fruit corn
Grain	Biscuit	A flavour reminiscent of sweet, wheat smell, associated with biscuit flavours	Grain cracker
	Cereal	A flavour reminiscent of cereal	Oatmeal
	Barley tea	A flavour reminiscent of barley tea	Barley tea
Green	Grass	A flavour reminiscent of freshly mowed grass	Lawn
	Cucumber	A flavour reminiscent of fresh, green, associated with cucumber	Cucumber
	Plantule	A flavour reminiscent of fresh, green, musty, humid, associated with barley sprouting	Germinated barley
	Dry grass	A flavour reminiscent of grass dried in the sun	Dry grass
Other	Sweet	A taste factor stimulated mainly by sucrose	Sucrose solution

Sweet aftertaste	The sweetness which lasts more than 10s	Liquid sugar
Wine sour	The sour from red wine	Red wine
Espresso sour	The sour from espresso	Espresso
Sour	A taste factor stimulated mainly by citric acid	Citric acid solution
Black coffee bitter	The bitterness from the coffee	Black coffee
Bitter	A taste factor stimulated mainly by quinine and caffeine	Quinine hydrochloride solution
Lightness	A quality that liquid flowing easily	Water
Astringency	A quality that causes oral tissue to become tight	Alum solution
Opaque	Not clear enough to see through or allow light through	Espresso
Consistency	The consistency of the liquid	Coffee

"AIH SRM Number Scale" from craft beer equipment retailer "Adventures in Homebrewing" (https://www.homebrewing.org/SRM-Beer-Colour-Scale_ep_81-1

Supplementary Table 2. Rate-all-that-apply anaysis for the six brewing malt samples by the laboratory panel (*p*<0.05).

Sample	S6	S7	S11	S14	S17	S20	Significance[1]
Toast	3.00	0.00	1.83	0.00	0.00	0.00	0.005
Caramelized sweet potato	3.00	0.00	4.17	4.67	0.00	0.00	0.006
Honey	1.83	0.00	1.83	0.00	0.00	0.00	0.006
Red dates	1.83	2.17	3.50	3.33	0.00	0.00	0.007
Cereal	2.67	0.00	0.00	0.00	0.00	0.00	0.005
Caramel	1.67	0.00	2.67	0.00	0.00	0.00	0.005
Biscuit	0.00	3.00	0.00	0.00	0.00	0.00	0.005
Roasted	0.00	2.50	0.00	0.00	4.50	4.17	0.006
Smoked	0.00	0.00	0.00	1.83	3.83	4.00	0.006
Burnt	0.00	2.67	3.00	3.00	0.00	0.00	0.011
Roasted nut	0.00	3.67	0.00	0.00	1.83	0.00	0.005
Hazel	0.00	0.00	2.00	0.00	0.00	0.00	0.005
Almond	0.00	0.00	0.00	2.17	0.00	0.00	0.005
Black chocolate	0.00	0.00	0.00	0.00	4.17	3.67	0.006
Coffee	0.00	0.00	0.00	0.00	3.17	3.83	0.005
Sour	0.00	2.33	2.50	4.00	4.33	4.00	0.013
Sweet	3.67	2.00	3.67	3.00	0.00	0.00	0.008
Bitter	0.00	2.50	0.00	3.17	4.67	4.83	0.006

"1" Rate-all-that-apply analysis scores of malt samples are significantly different ($p < 0.05$) using Fisher's least significant difference (LSD) test.

Supplementary Table 3. The volatile compounds and their contents in brewing malts.

No.	CAS	Compounds	Retention Index	Contents（ug/kg）																	
				S1	S2	S3	S5	S7	S8	S9	S10	S11	S12	S13	S14	S15	S16	S18	S19	S20	S21
1	78-84-2	Isobutyraldehyde	552	-	-	-	16.45	7.04	26.77	20.75	5.41	4.42	7.04	7.31	16.99	10.56	-	-	-	-	-
2	590-86-3	Isovaleraldehyde	652	10.26	10.14	12.87	175.02	40.28	67.22	59.56	62.98	78.94	38.78	50.28	39.5	104.06	4.77	9.94	6.44	3.88	2.67
3	96-17-3	2-Methylbutyraldehyde	662	-	-	-	129.16	78.95	112.93	98.84	44.26	74.77	29.32	32.5	41.28	67.86	12.71	22.15	17.3	7.75	6.96
4	110-62-3	Valeraldehyde	699	3.59	7	5.34	9.95	1.85	6.62	9.5	-	-	4.4	-	-	-	-	8.77	-	3.48	9.27
5	624-92-0	Dimethyl disulfide	746	-	-	-	-	-	3.52	-	-	-	1.95	-	-	-	-	-	-	-	-
6	66-25-1	Hexanal	800	21.76	12.71	10.06	32.85	15.21	17.19	23.98	114.72	10.33	22.32	3.19	8.04	52.56	22.78	115.42	11.43	112.97	10.03
7	109-08-0	2-Methylpyrazine	831	-	-	-	-	-	-	-	-	-	-	-	-	-	-	-	21.18	-	-
8	98-01-1	Furfural	833	-	-	-	32.21	202.4	79.81	113.01	34.03	8.16	56.53	76.53	304.93	649.48	1061.68	1061.01	1108.67	789.99	888.49
9	98-00-0	Furfuryl alcohol	859	-	-	-	-	-	7.76	-	-	-	-	-	-	6.8	-	-	-	-	-
10	28467-88-1	2-Methyl-2-hexenal	884	-	-	-	-	-	-	3.74	-	-	-	-	-	-	-	-	-	-	-
11	100-42-5	Styrene	893	-	-	-	-	-	-	-	4.7	-	-	-	-	-	-	-	-	-	-
12	106-42-3	P-Xylene	865	-	-	-	-	-	-	-	5.87	-	3.34	-	-	-	-	-	-	-	-
13	110-43-0	2-Heptanone	891	-	-	-	-	-	-	-	-	-	-	-	-	-	-	-	-	-	-
14	111-71-7	Heptaldehyde	901	-	-	-	-	-	-	-	5.46	-	-	-	-	-	-	-	-	16.52	-
15	1192-62-7	2-Acetylfuran	911	-	-	-	-	19.07	12.53	30.72	-	-	-	8.21	35.2	47.68	21.03	69.89	39.7	15.86	-
16	96-48-0	Gamma Butyrolactone	915	-	-	-	-	-	-	-	-	-	-	-	-	14.84	-	-	-	-	-
17	19549-83-8	2,6-Dimethyl-3-heptanone	985	-	-	-	-	-	-	-	-	7.11	4.86	3.3	-	-	-	-	-	-	-
18	100-52-7	Benzaldehyde	962	9.1	5.29	15.57	197.79	34.05	64.36	63.17	46.96	42.32	27.08	20.47	20.81	44.49	116.86	221.69	31.67	63.51	22.02
19	620-02-0	5-Methyl furfural	965	-	-	-	-	65.08	19.99	30.53	-	-	5.99	2.88	45.33	7.56	485.82	934.8	542.26	384.87	446.22
20	3391-86-4	1-Octen-3-ol	980	31.97	-	-	-	-	-	-	12.23	-	-	-	-	-	-	-	-	-	-
21	3777-69-3	2-Pentylfuran	993	53.99	49.66	37.8	14.06	8.41	7.56	10.57	14.72	5.14	3.22	-	5.98	17.88	25.23	78.83	8.89	43.27	13.43
22	13925-03-6	2-Ethyl-6-methylpyrazine	1003	-	-	-	-	8.84	8.13	7.7	-	-	-	-	-	-	42.5	-	62.61	-	25.37
23	109-52-4	Valeric acid	903	-	-	-	-	-	-	-	-	-	-	-	-	-	-	-	-	-	-
24	1003-29-8	2-Pyrrolylcarboxaldehyde	1015	-	-	-	-	-	-	-	-	-	-	-	-	-	17.62	14.26	27.14	11.06	1.34
25	142-62-1	Hexanoic acid	990	-	-	-	-	-	-	-	-	-	-	-	-	-	-	-	-	-	-
26	104-76-7	2-Ethylhexanol	1030	37.12	30.34	35.84	-	9.04	6.04	10.42	33.34	3.25	5.17	6.53	7.96	37.46	14.93	-	16.01	114.63	8.59
27	2463-77-6	2-Undecenal	1367	-	-	-	-	-	-	-	-	-	-	-	-	-	19.86	-	-	-	-

28	122-78-1	Phenylacetaldehyde	1045	44.95	47.39	37.71	475.27	74.9	193.96	113.99	83.38	86.72	38.51	36.64	21.68	91.5	10.93	-	7.49	9.28	-
29	2548-87-0	Trans-2-octenal	1060	7.83	5.2	6.02	2.88	9.58	11.32	15.12	27.74	1.92	5.64	1.41	4.54	15.67	24.33	63.33	11.9	42.23	15.56
30	1072-83-9	2-Acetyl pyrrole	1064	-	-	-	-	9.24	16.14	14.89	3.17	20.12	10	10.52	9.58	14.68	-	23.61	15.16	-	-
31	13925-07-0	3,5-Dimethyl-2-ethylpyrazine	1084	-	-	-	-	5.19	14.18	5.83	7.17	0.78	1.42	-	-	-	17.52	12.18	8.88	-	16.24
32	1120-21-4	Undecane	1100	560.44	541.9	573.9	589.1	142.81	225.07	218.43	249.05	224.97	193.78	206.7	-	395.19	191.49	543.59	177.71	231.99	157.14
33	124-19-6	Nonanal	1104	21.54	20.73	16.65	78.41	15.38	38.41	47.47	84.84	28.21	17.18	42.89	19.41	73.36	115.64	39.46	67.73	69.24	54.46
34	35158-25-9	2-Isopropyl-5-methyl-2-hexenal	1106	-	-	-	19.45	-	-	-	33.6	19.42	12.83	16.08	13.32	30.98	-	-	-	-	-
35	18829-56-6	Trans-2-nonenal	1162	32.85	12.45	-	-	-	-	-	13.98	-	-	-	-	-	-	-	-	-	-
36	118-71-8	Maltols	1100	-	-	-	-	-	-	-	7.8	12.08	6.25	13.05	89.43	26.97	10.22	42.7	12.08	16.64	8.21
37	75039-84-8	Trans--2-undecen-1-ol	-	-	-	-	-	-	-	-	-	-	5.03	-	-	-	-	-	-	-	-
38	13925-06-9	2-Methyl-3-isobutylpyrazine	1134	-	-	-	-	6.46	26.29	7.5	-	-	-	-	-	-	71.51	12.32	12.08	4.91	10.81
39	28564-83-2	2,3-Dihydro-3,5-dihydroxy-6-methyl-4(H)-pyran-4-one	1151	-	-	-	-	-	-	-	-	-	6.15	-	4.81	-	-	-	-	-	-
40	143-08-8	1-Nonanol	1173	-	9.78	-	-	-	-	-	-	-	-	-	-	-	-	-	-	-	-
41	13678-51-8	2-Furfuryl-5-methylfuran	1190	-	-	-	-	-	-	-	-	-	-	-	-	-	-	53.66	-	14.8	-
42	1438-94-4	1-Furfurylpyrrole	1187	-	-	-	-	4.46	7.65	6.73	-	-	-	-	2.35	-	32.69	61.41	25.83	23.02	27.2
43	112-31-2	Decyl aldehyde	1206	3.54	13.3	5.53	10.02	6.99	13.21	10.15	32.87	5.55	11	8.69	9.8	16.86	35.79	27.32	15.03	16.68	13.58
44	10042-59-8	2-Propylheptan-1-ol	-	30.16	26.42	27.91	21.02	38.08	46.63	26.41	48.36	31.69	8.45	15.56	65.85	48.75	105.78	-	52.36	73.89	31.51
45	1731-84-6	Methyl nonanoate	1225	-	-	-	-	-	-	-	-	8.93	-	-	-	-	-	-	-	-	-
46	67-47-0	5-Hydroxymethylfurfural	1233	-	-	-	-	-	-	-	-	-	-	-	11.21	-	-	-	-	-	-
47	91010-41-2	2-Isoamyl-6-methylpyrazine	1249	-	-	-	-	27.94	-	-	-	-	-	-	-	-	-	-	-	17.79	-
48	14360-50-0	2-hexanoylfuran	1239	-	-	-	-	-	-	-	-	-	-	-	3.92	-	-	-	-	-	-
49	4411-89-6	2-Phenyl-2-butenal	1279	-	-	-	41.49	15.04	42.04	22.26	22.68	25.05	6.99	13.41	22.26	22.61	-	-	-	-	-
50	2785-89-9	4-Ethyl-2-methoxyphenol	1282	-	-	-	-	-	-	-	-	-	-	-	-	-	21.39	54.93	22.7	26.79	26.12
51	540-07-8	Pentyl hexanoate	1287	-	-	-	-	-	-	-	-	-	-	-	-	-	-	-	-	-	-
52	25152-84-5	Trans,Trans-2,4-Decadien-1-al	1317	-	-	-	-	5.58	12.23	5.46	41.67	3.03	5.36	0.79	5.22	7.49	-	13.4	-	-	-
53	112-44-7	Undecanal	1307	-	-	-	-	-	-	-	3.92	-	-	-	-	-	-	-	-	-	-
54	18433-98-2	2,5-Dimethyl-3-isopentylpyrazine	1315	-	-	-	11.12	23.95	5.75	16.89	-	-	-	-	-	-	37.76	33.44	22.91	11.61	19.16
55	110-42-9	Methyl decanoate	1325	-	-	-	-	-	-	-	-	8.18	-	-	-	-	-	-	-	-	-

56	26643-91-4	4-Methyl-2-phenyl-2-pentenal	1383	-	-	-	21.72	10.3	29	17.94	13.89	19.73	7.96	10.84	18.41	11.61	-	-	-	-	-
57	112-54-9	Dodecyl aldehyde	1409	-	-	-	-	-	-	-	4.22	-	-	-	-	-	-	-	-	-	-
58	17283-81-7	Dihydro-beta-ionone	1433	-	-	-	-	-	-	-	-	-	-	-	-	-	-	12.06	-	-	-
59	3796-70-1	Geranylacetone	1453	-	-	-	7.38	9.79	4.21	4.43	19.38	2.41	1.72	3.03	4.83	3.04	6.41	11.85	10.91	4.09	4.69
60	689-67-8	6,10-Dimethyl-5,9-undecadien -2-one	1456	-	80.75	41.72	-	-	-	-	19.38	-	-	-	-	-	37.49	-	-	-	-
61	112-53-8	1-Dodecanol	1473	13.86	26.82	16.94	28.46	10.02	16.06	48.62	5.44	11.24	4.55	6.3	30.12	17.01	-	11.88	-	7.14	-
62	21834-92-4	Cocal	1486	-	-	-	53.59	18.22	45.92	29.76	11.06	31.63	3.39	24.11	26.52	24.07	12.87	9.14	10.41	1.88	4.36
63	96-76-4	2,4-Di-tert-butylphenol	1519	-	13.62	-	21.71	-	-	3.76	12.6	-	5.5	4.78	-	17.23	-	-	-	-	-
64	5129-56-6	Methyl 10-methylundecanoate	1471	-	-	18.54	-	-	-	-	-	-	-	-	-	-	-	-	-	-	-
65	111-82-0	Methyl laurate	1526	-	28.7	-	-	-	-	-	-	11.09	-	-	-	-	-	-	-	-	-
66	6846-50-0	2,2,4-Trimethyl-1,3-pentanedi ol diisobutyrate	1588	-	85.6	-	106.11	-	41.07	23.53	-	29.78	26.26	23.35	58.49	101.23	84.44	139.79	46.58	62.06	-
67	5129-58-8	Methyl isomyristate	1686	-	26.21	15.49	-	-	-	-	-	-	-	-	-	-	-	-	-	-	-
68	5444-75-7	Benzoic acid 2-ethylhexyl ester	1735	-	-	-	-	-	4.89	-	-	-	-	-	8.86	-	-	-	-	-	-
69	316249	Pentadecanal	1715	-	-	22.48	-	-	-	-	-	-	-	-	-	-	-	-	-	-	-
70	124-10-7	Methyl myristate	1725	209.59	199.33	178.76	47.38	-	12.85	6.89	-	19.73	-	-	-	-	-	-	-	-	-
71	120-51-4	Benzyl benzoate	1762	-	-	-	6.91	-	-	-	-	-	-	-	-	-	-	-	-	-	-
72	7132-64-1	Methyl pentadecanoate	1820	55.26	56.93	57.81	-	-	-	-	-	-	-	-	-	-	-	-	-	-	-
73	1120-25-8	Palmitoleic acid methyl ester	1899	131.87	283.22	155.94	-	-	-	-	-	12.95	-	-	-	-	-	-	-	-	-
74	112-39-0	Methyl palmitate	-	1624.42	908.19	1474.14	500.77	10.51	40.52	28.32	16.21	11.81	-	-	7.21	-	-	41.82	-	-	-
75	112-63-0	Methyl linoleate	2092	25.73	298.14	56.95	-	-	14.02	-	-	268.87	-	-	-	-	-	-	-	-	-
76	112-62-9	Methyl oleate	2091	99.4	87	94.37	-	-	-	-	-	66.62	-	-	-	-	-	-	-	-	-

"-" Not detected.

Supplementary Table 4. Descriptive anaysis for the 18 brewing malt samples by the laboratory panel ($p<0.05$).

Sample	S1	S2	S3	S5	S7	S8	S9	S10	S11	S12	S13	S14	S15	S16	S18	S19	S20	S21	Significance[1]
Baking	1.60	1.73	2.00	2.25	2.75	2.70	3.29	2.39	2.67	2.57	2.63	3.07	2.79	3.70	4.27	3.98	4.16	3.93	0.000
Smoky	0.00	0.00	0.00	0.32	0.37	0.53	1.21	0.54	0.87	0.71	0.40	1.64	0.43	2.93	3.00	2.74	3.50	3.07	0.000
Nutty	0.83	0.90	0.77	1.20	1.40	1.60	2.36	1.00	1.30	1.35	0.90	0.80	0.70	0.37	0.00	0.45	0.00	0.00	0.000
Friuty	3.17	3.10	2.80	2.78	1.08	1.40	1.57	2.43	2.10	1.79	1.30	1.20	1.30	0.43	0.00	0.35	0.00	0.00	0.000
Caramel	1.07	1.30	0.73	2.35	1.48	2.35	2.43	2.29	2.13	2.21	3.60	4.21	3.64	0.93	1.27	0.98	1.00	0.73	0.000
Grain	1.07	1.07	0.60	1.56	0.62	0.98	1.11	1.04	1.47	1.11	1.07	0.90	1.04	0.00	0.00	0.41	0.00	0.00	0.000
Green	4.00	3.87	4.13	3.00	2.47	2.63	2.20	2.68	1.93	1.70	1.20	0.93	1.43	0.71	0.75	0.61	0.71	0.65	0.000

"1" Descriptive analysis scores of malt samples are significantly different ($p < 0.05$) using Fisher's least significant difference (LSD) test.
